# Biomechanical and clinical evaluations of superior capsular reconstruction using autologous tendon for irreparable rotator cuff tear

**DOI:** 10.3389/fbioe.2026.1801212

**Published:** 2026-05-13

**Authors:** Xiping Jiang, Xiaofeng Zhang, Pan Liu, Qi Chen, Chenrui Yuan, Chenkai Li, Wencai Liu, Wei Song, Xin Wang, Weilin Yu, Qingxiang Hu, Yaohua He

**Affiliations:** 1 Department of Orthopedics, Shanghai Sixth People’s Hospital Affiliated to Shanghai Jiao Tong University School of Medicine, Shanghai, China; 2 Department of Orthopedics, Jinshan District Central Hospital affiliated to Shanghai University of Medicine & Health Sciences, Shanghai, China; 3 Department of Orthopedics, Sichuan Provincial People’s Hospital, School of Medicine, University of Electronic Science and Technology of China, Chengdu, China

**Keywords:** autologous tendon, biomechanics, irreparable rotator cuff tears, peroneus longus tendon, superior capsular reconstruction

## Abstract

**Introduction:**

To evaluate the biomechanical and clinical outcomes of superior capsular reconstruction (SCR) with autologous tendon (AT) for the irreparable rotator cuff tears (IRCTs).

**Methods:**

Ten fresh-frozen human upper limb specimens were used to create three models (intact, rotator cuff-deficient, and reconstruction). Superior translation of the humeral head and subacromial pressure were measured at different abduction angles under standardized loading. Mechanical comparability between tendons was also evaluated by measuring cross-sectional area and basic tensile properties. For clinical evaluation, a prospective case series of nine patients with IRCTs tears who underwent SCR with AT was conducted. Shoulder joint range of motion, American Shoulder and Elbow Surgeons (ASES), and Constant-Murley (CM) were recorded before surgery, at 6 months and 12 months after surgery. Magnetic resonance imaging (MRI) was performed preoperatively, the day after surgery, and at 6 months.

**Results:**

The reconstruction reduced superior translation of the humeral head and subacromial pressure compared with the rotator cuff-deficient condition. No significant differences were observed in basic mechanical properties between the tested tendons. ASES scores improved significantly at both 6 and 12 months after surgery, whereas Constant score did not remain statistically significant in the 12-month pairwise comparison after adjustment for multiple comparisons. No significant differences were observed in shoulder joint range of motion. MRI showed 6/9 (67%) patients had reduced thickness of the implanted peroneus longus tendon (PLT) graft at 6 months postoperatively, while the PLT grafts of the other patients remained intact.

**Conclusion:**

SCR with AT may help restore superior glenohumeral stability under controlled biomechanical conditions and shows promising preliminary clinical results in patients with IRCTs.

## 1 Introduction

Irreparable rotator cuff tears (IRCTs) are defined as cuff lesions that cannot be repaired to the footprint region using conventional repair techniques (Oh et al., 2018; Schmidt et al., 2015). Superior capsular reconstruction (SCR) is a novel strategy for IRCTs first reported by Mihata et al. (2013). It uses a graft patch, with one side fixed to the superior rim of the glenoid and the other anchored to the greater tuberosity (Claro and Fonte, 2023). Biomechanical experiments have demonstrated that SCR can restrain humeral head superior translation, reduce subacromial pressure, and prevent graft abrasion, underscoring the positive role of SCR in treating IRCTs (Mihata et al., 2012; Mihata et al., 2016a; Mihata et al., 2016b).

In clinical practice, the grafts for SCR mainly include autologous fascia lata and allogenic dermal matrix (Milano et al., 2020; Zhao et al., 2022). Still, there is no consensus on graft choice. Using autologous fascia lata as a graft involves postoperative complications in the donor site (Wheatcroft et al., 1997). When using an allogenic dermal matrix, the biological healing is poor, and the risk of graft retear is high (Pennington et al., 2018). Therefore, it is clinically important to identify a graft that reduces postoperative complications in the donor site and improves IRCTs outcomes. Autologous tendon (AT) could be harvested through a mini-incision, resulting with a low complication rate and a high healing rate (Rhatomy et al., 2019; Rhee et al., 2008), which can be a proper candidate for SCR. Alcid et al. performed anterior shoulder capsule reconstruction using autologous peroneus longus tendon (PLT) for recurrent shoulder dislocations, suggesting the feasibility of the technique (Alcid et al., 2007).

This study proposed the biomechanical and clinical evaluations of SCR using autologous tendon for IRCTs. For the biomechanical aspect, this study explored the superior stability of the glenohumeral joint and subacromial contact between the graft and the acromion. For the clinical aspect, we investigated the effectiveness of using PLT as the SCR implant for IRCTs treatment. It was hypothesized that SCR with autologous AT could restrict the superior translation of the humeral head, reduce subacromial pressure, and restore the shoulder function for IRCTs patients.

## 2 Materials and methods

### 2.1 Specimen preparation

Ten fresh frozen specimens were provided by Shanghai Jiao Tong University School of Medicine. All cadaveric specimens were screened and excluded if there were rotator cuff tears, previous surgery, fractures, or structural abnormalities affecting shoulder biomechanics. Due to limited specimen availability, all eligible specimens were included. Prior to constructing the models, the shoulder specimens were dissected. After removing the skin and superficial fascia, the anterior serratus, pectoralis minor, and rhomboid muscles were removed. The deltoid, supraspinatus, subscapularis, infraspinatus, and teres minor muscles were preserved. The insertion points of these muscles on the humerus were protected. The tendon portions were sutured in a Krackow manner.

### 2.2 Model construction

**FIGURE 1:**
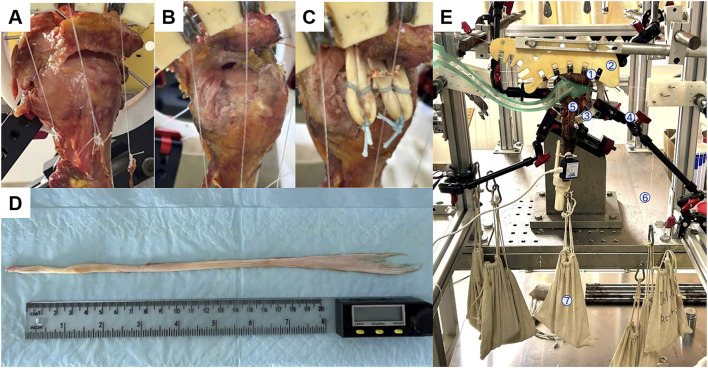
Shoulder models and biomechanical shoulder jig. **(A)** Intact shoulder joint. **(B)** IRCT. **(C)** Modified SCR using AT; **(D)** FDP tendon used as autologous tendon graft. **(E)** Biomechanical shoulder jig: ①Pulley system; ②Deltoid plate; ③Ring fixator; ④Ring holder; ⑤Upper extremity specimen; ⑥Force line; ⑦Weights.

**FIGURE 2:**
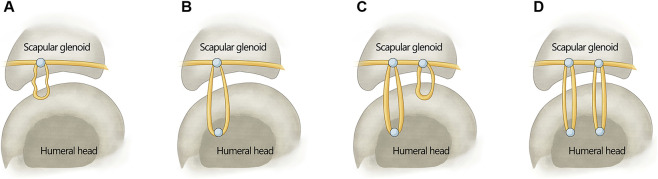
The illustration of SCR using AT for IRCTs repair. **(A)** Form the first Type “O” structure. **(B)** O-shaped structure was fixed on the greater tuberosity of the humerus. **(C)** A second O-shaped structure was formed. **(D)** A second a second O-shaped structure was fixed.

In this study, three models were constructed (Table 1). Model A was a normal shoulder joint with intact rotator cuffs (Figure 1A). Model B was the IRCTs condition, with the supraspinatus muscle and superior capsule removed (Figure 1B). Model C denoted the SCR with tendon transplantation scenario (Figure 1C). In this study, the flexor digitorum profundus tendon (FDP) was harvested as an autograft (Figure 1D). The final length of the tendon can be adjusted according to the defect area when the glenohumeral joint was abducted at 30°. As the tendon was fixed in several O-shaped structure manner, the length of the tendon should be preserved as much as possible. Then, the tendon stump was cut and sutured on both sides. The bone bed of the superior glenoid and humeral head was prepared with an arthroscopic burr. Taking the right shoulder as an example, two suture anchors (3.0 × 16 mm, non-absorbable belt rivet SAPC01, Delta, China) with double lines were inserted in the 10 and 11 o’clock directions of the scapula. The tendon was inserted into the joint cavity through the approach below the acromioclavicular joint, and the free tendon was loosely pull to the 10 o’clock position of the scapula to fix it with a suture anchor (Figure 2A). The free tendon was pulled behind the greater tuberosity and reserved a suitable length. The free tendon was fixed with the second suture anchored at 10 o’clock (Figure 2B). The first type “O” structure was then formed. The length of the “O” structure was about 3.5–4.0 cm. By stretching the O-shaped structure, the length was increased by about 0.5 cm. We fixed O-shaped structure on the greater tuberosity of the humerus with transosseous-equivalent technique fixation. Then, we moved the free tendon to the 11 o’clock position of the labrum and fixed it with suture anchor (Figure 2C). Following the previous folding method, a second O-shaped structure was generated. We pulled the tendon again to form two strands of tendon fixation in front of the greater tuberosity on the upper labrum (Figure 2D). The posterior tendon was sutured to the end of the posterior rotator cuff tissue, and the excess free muscle was removed in the anterior region. The graft was fixed when the glenohumeral joint was abducted at 30°.

**TABLE 1:** Experimental scenarios.

Conditions	Model	Deltoid	Supra	Infra and TM	Subs	GJP
Normal shoulder joint (control)	A	Balance load: 40 N	10 N	10 N	10 N	0°, 30°, 60°ABD
		Superior translation load: 80 N				
Irreparable rotator cuff tear	B	Balance load: 40 N	0	10 N	10 N	0°, 30°, 60°ABD
		Superior translation load: 80 N				
SCR with adjustable free tendon transplantation	C	Balance load: 40 N	0	10N	10N	0°, 30°, 60° ABD
		Superior translation load: 80 N				

Supra: supraspinatus; Infra: infraspinatus; TM: teres minor; Subs: subscapularis; GJP: glenohumeral joint position; ABD: abduction.

### 2.3 Testing platform and testing measurement

The biomechanical testing platform for the shoulder joint used in this study was built based on designs from previous research (Hu et al., 2018). It consisted of several key components: a scapular fixation module, a weight-loading module, a deltoid muscle pulley module, and a deltoid force-loading module. In addition, it was equipped with measurement devices, including a contact pressure testing system (Tekscan model 4205, Boston, MA, United States), an abduction angle sensor (WT901WIFI, WitMotion, Hong Kong, China), and a coordinate measuring machine (Microscribe3dMLX, Revware, Inc., NC, United States) to capture relevant physical parameters accurately (Figure 1E).

The shoulder specimens were mounted to the scapular fixation module with the scapula positioned at a 20° anterior tilt in the sagittal plane. Each model was tested when the shoulder was abducted at 0°, 30°, and 60°. The rotator cuffs were loaded according to the sectional area of muscles: 10 N for supraspinatus, 10 N for infraspinatus and teres minor, and 10 N for subscapularis. For models B and C, the supraspinatus was not loaded. Two deltoid loadings were used to measure the superior translation of the humeral head: balance and superior translation load (Table 2). Under balance load, the vertical distance between the humeral head and acromion was measured using Microscribe3dMLX. Subsequently, the vertical distance was measured again under a superior translation load. The difference between the two measurements represented the humeral head’s superior translation distance. For peak contact pressure measurement, the Tekscan pressure sensor was carefully placed beneath the coracoacromial arc under the superior translation load. Then, the peak contact pressures were measured. The deltoid loading conditions (40 N for balance load and 80 N for superior translation load) were selected based on previously established cadaveric shoulder biomechanical models (Mihata et al., 2012; Mihata et al., 2016a). These loading magnitudes have been used to simulate physiologically relevant conditions *in vitro* while avoiding excessive tissue damage or non-physiological joint behavior. The 40 N load was applied to represent a balanced resting condition of the shoulder, allowing evaluation of baseline joint alignment. The 80 N load was used to simulate an increased superiorly directed force generated by the deltoid, thereby inducing superior translation of the humeral head and enabling assessment of joint stability under stress conditions.

**TABLE 2:** Humeral head upward displacement.

Glenohumeral abduction	Translation, cm		
	Model A	Model B	Model C
0°	0.17 ± 0.02 (0.16–0.19)	0.48 ± 0.05 (0.44–0.52)^*^	0.20 ± 0.02 (0.18–0.22)^#^ ^,^ ^†^
30°	0.14 ± 0.02 (0.13–0.16)	0.34 ± 0.04 (0.31–0.37)^*^	0.15 ± 0.02 (0.13–0.17)^†^
60°	0.11 ± 0.02 (0.10–0.13)	0.21 ± 0.02 (0.20–0.22)^*^	0.12 ± 0.02 (0.11–0.13)^†^

Model A: normal shoulder joint; model B: irreparable rotator cuff tear; model C: modified superior capsular reconstruction.

^*^ A significant difference was found compared with model A.

^†^ A significant difference was found compared with model B.

^#^ A significant difference was found compared with model A.

### 2.4 Mechanical testing and histological analyses of FDP tendon and PLT

**FIGURE 3:**
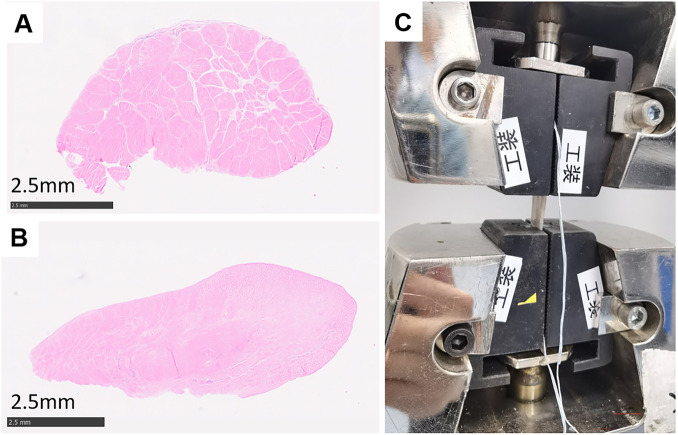
Mechanical testing and histological analyses of FDP and PLT. **(A)** H&E staining of the cross-section of FDP tendon. **(B)** H&E staining of the cross-section of PLT. **(C)** Universal mechanical testing machine loaded with tendon.

FDP and PLT were harvested, with three samples included in each group. Half-width PLT was used, consistent with the graft preparation applied in the clinical procedure. For each specimen, a small portion of the tendon was excised for histological analysis. The samples were processed for hematoxylin and eosin (H&E) staining (Figures 3A,B), and cross-sectional area was measured using histological sections. The remaining tendon tissue was prepared for biomechanical testing (Figure 3C). Both ends of each tendon were reinforced using a braided suture technique to prevent slippage during testing. Mechanical testing was performed using a universal testing machine (UTM5305SYXL, SUNS Technology Stock Co., Ltd., Shenzhen, China). A movement rate of 300 mm/min was administered to each specimen until failure. The tendons were loaded to failure, and the maximum load was recorded. Stiffness was calculated from the initial 5%–10% increase region of the force-displacement curve. Maximum stress was subsequently calculated based on the measured cross-sectional area.

### 2.5 Surgical procedure and rehabilitation

A prospective case series of nine patients with IRCTs who underwent SCR with PLT was conducted. IRCTs were primarily defined based on tendon retraction. Specifically, tears were considered irreparable when the torn tendon was retracted beyond the glenoid rim and could not be mobilized back to its anatomical footprint despite adequate release during surgery. This intraoperative criterion was used as the main determinant of irreparability. Preoperative magnetic resonance imaging (MRI) findings, including tendon retraction and fatty degeneration, were used as supportive evidence but were not applied as strict inclusion thresholds. Patients were included if they had symptomatic IRCTs with persistent pain and functional impairment after failed conservative treatment. Given that this surgical technique is relatively novel and has not yet been widely adopted, all consecutive patients meeting these criteria during the study period were included. Patients were excluded if they had advanced glenohumeral arthritis, active infection, or severe comorbidities that could affect surgical outcomes. For clinical evaluation, shoulder joint range of motion, American Shoulder and Elbow Surgeons (ASES), and Constant-Murley (CM) were recorded before surgery, at 6 and 12 months after surgery. Clinical assessors were independent of surgical team and blinded to study objectives. MRI was performed preoperatively, the day after surgery, and at 6 months postoperatively.

All the surgeries were conducted by a single surgeon (Y. H.). The arthroscopic procedure was performed with the patient in the lateral decubitus position under general anesthesia and an interscalene block. Initially, a standard posterior portal was established to access the shoulder joint. Subsequently, an anterior portal was created for further arthroscopic maneuvering. A comprehensive examination of the glenohumeral joint was conducted through both portals, including an assessment of the long head of biceps tendon (LHBT) within the joint. The arthroscope was then directed into the subacromial space, and a lateral portal was established to perform acromioplasty and bursectomy. Additionally, Kim’s portal, positioned 2 cm lateral to the line extending from the back-lateral edge of the clavicle to the posterior-lateral edge of the acromion, was utilized as the primary viewing portal for evaluating and addressing torn rotator cuff tendons (Chernchujit and Parate, 2017).

**FIGURE 4:**
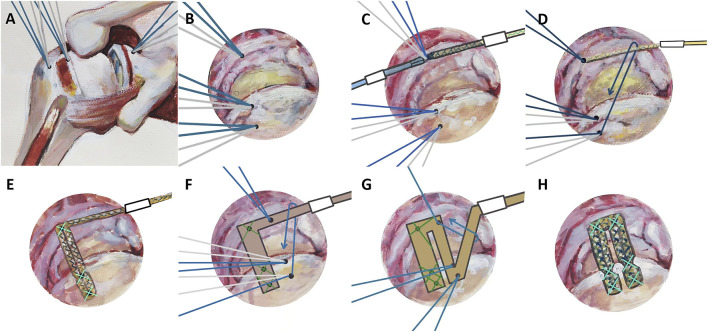
The illustration of SCR using PLT for IRCTs repair. **(A,B)** A double-threaded glenoid rim anchors were placed, and two double-threaded suture anchors were placed at the greater tuberosity. **(C)** The thickened end of the PLT was fixed at the 10 o’clock position via the glenoid rim anchor. **(D)** A free suture guided PLT to the posterior side of the greater tuberosity. **(E)** PLT was fixed to the greater tuberosity via two suture anchors. **(F)** The free end of PLT was fixed to the 11 o’clock position of the glenoid rim anchor. **(G)** The PLT tendon was fixed to the great tuberosity using a free suture and two suture anchors. **(H)** Overview of the SCR using PLT.

If the evaluation showed IRCTs with inadequate quality or insufficient length for repositioning of the LHBT, the ipsilateral PLT was harvested as the autograft for IRCTs repair. A graft was prepared using half of the width of PLT. The graft was approximately 25 cm long and 6–10 mm wide, fully woven, with both ends leaving approximately 10 cm of suture. Two double-threaded glenoid rim anchors (210811, Johnson & Johnson, NJ, United States) were placed at 10 and 11 o’clock positions (for the right shoulder) or 1 and 2 o’clock positions (for the left shoulder) (Figures 4A,B). The harvested PLT was introduced into the subacromial space through a portal below the acromioclavicular joint. The thickened end of the PLT was grasped from the posterior portal and pulled to the glenoid rim anchor at the 10 o’clock position for suture fixation (Figure 4C). A free suture was passed through the PLT and guided it to the posterior side of the greater tuberosity (Figure 4D). The second suture from the 10 o’clock position was passed through the free tendon and temporarily fixed with a knot pusher. The tendon was approximately 1 cm from the articular cartilage of the humeral head at the greater tuberosity. One suture anchor (AR-2324BCC, Arthrex, FL, United States) or two suture anchors (222295, Johnson & Johnson, NJ, United States) were used for fixation at the greater tuberosity (Figure 4E). The temporary fixation of the suture at the 10 o’clock position was then completely fixed to secure the tendon. Subsequently, the free end of the PLT was shifted to the 11 o’clock position of the glenoid rim anchor for suture fixation (Figure 4F). The tendon was folded back again as previously described, fixing it in two places at the superior rim and in front of the greater tuberosity (Figure 4G). For most patients, two folds were adequate (Figure 4H), but additional folds could be performed if necessary. The free suture on the anchor at the greater tuberosity was applied to fix the PLT again. The posterior part of the folded PLT and the remanent posterior rotator cuff tendon were sutured end-to-end, and the excess free end of PLT in front was excised. The anchor number and configuration in this procedure were standardized. The surgical video of this procedure has been published (Wan et al., 2025).

A shoulder abduction brace was applied for 6 weeks post-surgery. All patients received standardized rehabilitation postoperative instructions. Pendulum exercises were initiated within the first 3 weeks after surgery, followed by pulley exercises and passive shoulder movements at 4 weeks postoperatively. Active exercises began at 3 months after surgery, and most patients were cleared for all sports activities by 6 months post-surgery. In cases of slow recovery, an assistant physical therapist provided additional support.

### 2.6 Statistical analysis

Statistical analysis was conducted using SPSS 27.0 (IBM, Armonk, NY, United States). A power analysis was routinely performed based on superior translation by using Power Analysis and Sample Size software version 11.0 (PASS, NCSS Statistical Software, LLC, Utah, United States). At least eight cadavers should be adopted to provide >80% power for the tests. For biomechanical outcomes, statistical analyses were performed using a two-way repeated-measures analysis of variance (RM-ANOVA), with model (A, B, and C) and glenohumeral abduction angle (0°, 30°, and 60°) as within-subject factors, because each cadaveric specimen was tested repeatedly under all experimental conditions. Separate RM-ANOVA models were constructed for humeral head superior translation and peak subacromial pressure. When a significant interaction or main effect was detected, *post hoc* pairwise comparisons between models at each abduction angle were performed using paired t-tests with Bonferroni correction. Continuous data are presented as mean ± standard deviation (SD), together with 95% confidence intervals (CI) where appropriate. Effect sizes for RM-ANOVA were reported as partial eta squared (ηp^2^), and effect sizes for pairwise comparisons were expressed as Cohen’s dz. For mechanical properties comparison of FDP and PLT, due to the small sample size, non-parametric statistical analysis (Mann-Whitney U test) was used to compare the mechanical properties between groups. Data are presented as mean ± SD. A two-sided P value <0.05 was considered statistically significant.

For the clinical cohort, sample size adequacy was evaluated based on the primary clinical endpoint, defined as the change in ASES score from preoperative assessment to postoperative 12 months. Because the study used a within-subject repeated-measures design, each patient served as his or her own control, which reduced inter-individual variability and improved statistical efficiency. Based on the observed paired change in ASES score in the present cohort (mean improvement, 29.6 points; SD of paired differences, 10.9 points), the standardized paired effect size (Cohen’s dz) was 2.71. Using a two-sided alpha level of 0.05% and 80% power for a paired comparison, the minimum required sample size was 4 patients. Therefore, the inclusion of 9 patients was considered sufficient for detecting a clinically meaningful improvement in the primary functional outcome. Nevertheless, the study may still be underpowered for smaller effects in secondary endpoints such as range-of-motion variables, and the clinical findings should therefore be interpreted as exploratory. For clinical outcomes, they were analyzed using non-parametric repeated-measures methods because of the small sample size (n = 9) and the within-subject longitudinal design. Differences among the three time points (preoperative, postoperative 6 months, and postoperative 12 months) were first assessed using the Friedman test for each clinical parameter. When appropriate, *post hoc* pairwise comparisons between postoperative time points and baseline were performed using the Wilcoxon signed-rank test. To account for multiple comparisons within each endpoint, P values for pairwise analyses were adjusted using the Holm method. A two-sided P value <0.05 was considered statistically significant.

## 3 Results

### 3.1 Characteristics of the specimens

Among the 13 specimens received for this experiment, three with rotator cuff injuries were excluded. Among the ten remaining specimens, four were from males and six from females. The average age of the donors was 72.6 years (range, 60–87 years)

### 3.2 Superior translation of the humeral head

Two-way repeated-measures ANOVA demonstrated significant main effects of model (F (2,18) = 436.953, P < 0.001, ηp^2^ = 0.980) and abduction angle (F (2,18) = 171.226, P < 0.001, ηp^2^ = 0.950), as well as a significant model-by-angle interaction (F (4,36) = 89.838, P < 0.001, ηp^2^ = 0.909). Post hoc analyses showed that model B had significantly greater superior translation than model A at all tested angles (all Bonferroni-corrected P < 0.001). Compared with model B, model C significantly reduced superior translation at 0°, 30°, and 60° abduction (all Bonferroni-corrected P < 0.001). Model C showed slightly greater translation than model A at 0° abduction (mean difference: 0.027 cm, 95% CI: 0.007–0.047 cm, corrected P = 0.033), whereas no significant differences between models A and C were observed at 30° or 60° abduction (Table 2).

### 3.3 Peak subacromial pressure

Two-way repeated-measures ANOVA demonstrated significant main effects of model (F (2,18) = 1097.757, P < 0.001, ηp^2^ = 0.992) and abduction angle (F (2,18) = 228.450, P < 0.001, ηp^2^ = 0.962), together with a significant interaction effect (F (4,36) = 41.810, P < 0.001, ηp^2^ = 0.823). Post hoc analyses showed that model B had significantly higher peak subacromial pressure than model A at all tested angles (all Bonferroni-corrected P < 0.001). Compared with model B, model C significantly reduced peak subacromial pressure at 0°, 30°, and 60° abduction (all Bonferroni-corrected P < 0.001). No significant difference in pressure was observed between models A and C at 0° abduction (corrected P = 1.000), whereas significant differences were found at 30° and 60° abduction (Table 3).

**TABLE 3:** Peak subacromial pressure.

Glenohumeral abduction	Pressure, MPa		
	Model A	Model B	Model C
0°	0.48 ± 0.03 (0.46–0.51)	1.18 ± 0.08 (1.12–1.24)^*^	0.49 ± 0.07 (0.44–0.54)^†^
30°	0.58 ± 0.07 (0.54–0.63)	1.03 ± 0.09 (0.97–1.09)^*^	0.67 ± 0.05 (0.64–0.70)^*^ ^,^ ^†^
60°	1.04 ± 0.14 (0.94–1.13)	1.47 ± 0.04 (1.45–1.50)^*^	0.81 ± 0.05 (0.78–0.85)^#^ ^,^ ^†^

Model A: normal shoulder joint; model B: irreparable rotator cuff tear; model C: modified superior capsular reconstruction.

^*^ A significant difference was found compared with model A.

^†^ A significant difference was found compared with model B.

^#^ A significant difference was found compared with model A.

### 3.4 Mechanical properties comparison between FDP and PLT

The mechanical properties of the FDP and PLT were compared. A half-width PLT was used, replicating the graft configuration applied in the clinical procedure. There were no statistically significant differences between the two groups in cross-sectional area (20.7 ± 2.0 vs. 21.7 ± 1.6 mm^2^, P = 1.000), maximum load (622.2 ± 85.6 vs. 730.7 ± 29.0 N, P = 0.200), maximum stress (29.94 ± 1.34 vs. 33.73 ± 1.23 MPa, P = 0.100), or stiffness (134.2 ± 47.0 vs. 103.5 ± 20.4 N/mm, P = 1.000) (Table 4).

**TABLE 4:** Mechanical properties comparison between FDP tendon and PLT.

Group & statistical analysis	Cross-sectional area (mm^2^, n = 3)	Maximum load (N, n = 3)	Maximum stress (MPa, n = 3)	Stiffness (N/mm, n = 3)
FDP	20.7 ± 2.0	622.2 ± 85.6	29.94 ± 1.34	134.2 ± 47.0
PLT	21.7 ± 1.6	730.7 ± 29.0	33.73 ± 1.23	103.5 ± 20.4
P Value	1.000	0.200	0.100	1.000

### 3.5 Patient characterization

All nine IRCTs patients who received SCR using PLT from April 2023 to July 2023 were included in this study. There were 4 males and 5 females, with an average age of 64 ± 6.4 years. The average duration of symptoms was 8.0 ± 7.2 months. The affected shoulder for all patients was the dominant side. Five, one, or three patients were involved with high, medium, or low labor force demands, respectively. The average BMI of these patients was 23.5 ± 3.3. None of the patients smoked or had diabetes, although four patients had hypertension. Two of the nine patients had a history of shoulder dislocation before surgery. The mean operative time was 141.1 ± 26.2 min. According to the Goutallier classication (Goutallier et al., 2003), preoperatively, five patients demonstrated Goutallier grade 2 fatty degeneration of the supraspinatus, while four patients were classified as Goutallier grade 3 fatty degeneration of the supraspinatus.

### 3.6 Clinical and radiological outcomes

**FIGURE 5:**
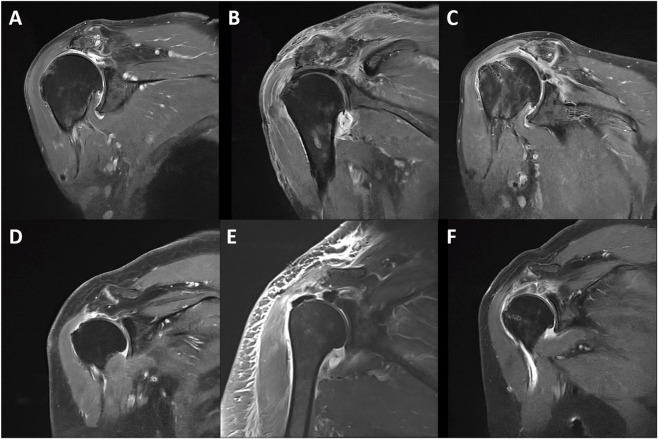
Representative MRI images preoperatively, at the day after surgery, and at 6 months postoperatively. **(A–C)** from one patient at different timepoints: **(A)** before surgery; **(B)** the day after surgery; **(C)** at 6 months follow-up. **(D–F)** from another patient at different timepoints: **(D)** before surgery; **(E)** the day after surgery; **(F)** at 6 months follow-up.

As shown in Table 5, Friedman testing demonstrated significant overall time-dependent changes in ASES score (χ^2^ = 15.600, P = 0.0004) and Constant score (χ^2^ = 11.556, P = 0.0031), whereas FE (P = 0.097), AB (P = 0.237), 0° ER (P = 0.105), and IR at back (P = 0.249) did not show significant overall differences across the three time points. Post hoc Wilcoxon signed-rank tests with Holm adjustment showed that ASES score was significantly improved at postoperative 6 months (adjusted P = 0.0391) and postoperative 12 months (adjusted P = 0.0078) compared with the preoperative level. Constant score showed improvement at postoperative 12 months, but this difference did not remain statistically significant after adjustment for multiple comparisons (adjusted P = 0.0781). No significant pairwise differences were found for FE, AB, 0° ER, or IR at back. No ankle weakness, sensory changes, or pain at the PLT donor site were reported at the 12-month follow-up. The two patients with a history of shoulder dislocation did not experience any recurrence. On qualitative MRI evaluation at 6 months postoperatively, 6/9 (67%) patients showed an apparent reduction in graft thickness or graft remodeling compared with the immediate postoperative images (Figures 5A–C), whereas the graft appearance remained relatively preserved in the remaining cases (Figures 5D–F). At 6 months postoperatively, no obvious changes in the degree of supraspinatus fatty degeneration were observed.

**TABLE 5:** Clinical outcomes of preoperative and postoperative follow-up periods.

ROM & functional scores	Preoperative (n = 9)	Postoperative 6 months (n = 9)	Postoperative 12 months (n = 9)	P Value (6 months vs. preoperative)	P Value (12 months vs. preoperative)
ROM					
FE (°)	165.8 ± 28.7	145.9 ± 51.8	166.8 ± 27.2	0.426	0.820
AB (°)	167.8 ± 27.3	159.8 ± 43.5	166.3 ± 30.6	0.641	0.945
0° ER (°)	40.6 ± 27.7	44.6 ± 21.8	53.7 ± 13.9	0.641	0.074
IR at back	5.0 ± 1.2	4.0 ± 2.1	4.5 ± 2.1	0.438	0.875
Scores					
ASES	52.1 ± 13.1	69.3 ± 20.3	81.7 ± 13.4	0.039	0.008
Constant	57.0 ± 8.6	63.4 ± 22.0	75.3 ± 16.0	0.426	0.078

FE: forward elevation; AB: abduction; ER: external rotation; IR: internal rotation. The levels of IR, at the back were converted to numerical data for analysis based on previously published study: 0 for sacral level, one to five for L5-L1, and 6-12 for T12-T6 (Yang et al., 2023).

## 4 Discussion

The most significant finding of this study is that AT may be used as a graft for SCR, limiting superior translation of the humeral head and decreasing subacromial pressure. The advantage of using AT is the elasticity of the closed loop formed by tendon. We utilize the elasticity and ultimate stretching of this tendon ring to fix it at the greater tuberosity, thus creating inward pressure on the humeral head. The key point of this technology is to utilize the elasticity of the tendon ring, not only to complete SCR, but also to apply additional stress to the humeral head.

In the past decade, SCR has played an important role in treating IRCTs, yielding favorable clinical outcomes (Mihata et al., 2018), but graft selection remains controversial. Initially, Mihata proposed using autologous fascia lata as a graft, but 57% of patients experienced complications in the donor site postoperatively (Hasegawa et al., 2021). Subsequently, scholars suggested using dermal allograft as a graft, yet with poor biological healing, reporting with a 38.2% failure rate (Jackson et al., 2023). Therefore, the best graft selection in clinical practice remains unclear. Hence, the authors believe that AT, commonly used in sports medicine, is a readily available and minimally invasive graft associated with low postoperative complications. Moreover, autogenous grafts are prone to better healing rates than allografts and therefore could be a potential material for SCR. Furthermore, inspired by the work of Alcid et al. who demonstrated that AT can be used to reconstruct the anterior capsule of the shoulder for treating anterior shoulder dislocations (Alcid et al., 2007), we proposed an adjustable tendon transplantation technique for SCR using AT to reconstruct the superior capsule of the shoulder.

The results of this study indicated that compared with IRCT, SCR with AT could limit the superior translation of the humeral head and reduce subacromial pressure. Previous biomechanical studies have reported similar results. Berthold et al. used the autologous LHBT for SCR in a posterior-superior massive IRCTs model (Berthold et al., 2024). In another study, Berthold et al. used autogenous hamstring tendons for SCR (Berthold et al., 2021). While these reconstruction configuration differed from the one used in our study, the results were consistent, indicating that SCR with AT can restore the superior stability of the glenohumeral joint and reduce subacromial contact pressure.

Clinically, the SCR with AT has advantages. First, it adopts several O-shaped structures, and the number of O-shaped structures can be determined based on the area of the defect. Generally, two to three O-shaped structures can be used side by side for the SCR, and excess tendons are removed. Second, the AT could be harvested with minimal donor site injury, allowing early rehabilitation. Third, the elasticity of the closed loop formed by the tendon can stretch this tendon ring to fix it at the greater tuberosity, thus creating inward pressure on the humeral head. For clinical evaluation of SCR with PLT, we observed that two patients with shoulder dislocations associated with IRCTs regained shoulder joint stability post-SCR with no recurrence of dislocation. The nine patients with IRCTs in this study had relatively good shoulder ROM preoperatively, with their primary concerns being unbearable shoulder pain, lack of upper limb strength, and shoulder instability. We did not observe improvement in ROM at the 12-month follow-up. However, clinical scores at 12 months postoperatively showed favorable results, suggesting that SCR using PLT may promote overall shoulder function recovery. Although the reconstructed superior capsule can restrain the upward migration of the humeral head to some extent, 6/9 (67%) patients showed reduced thickness of the implanted graft at 6 months postoperatively compared to the day after surgery. The absence of structural imaging at 12 months limits the ability to fully interpret graft remodeling and its long-term clinical implications, including graft thinning or potential failure. Extended follow-up with serial imaging will be necessary to better understand graft durability and its relationship to clinical outcomes.

In the present study, qualitative MRI evaluation at 6 months postoperatively suggested apparent graft thinning in a proportion of patients. This finding may be clinically relevant, as previous studies have reported that graft thinning following SCR may be associated with reduced mechanical integrity and less favorable clinical outcomes (Lee and Shin, 2024). From a biomechanical perspective, graft thinning may decrease the effective thickness and stiffness of the reconstructed superior capsule, thereby reducing its ability to resist superior translation of the humeral head. This could potentially compromise long-term joint stability and influence functional recovery. However, it should be noted that graft morphology in this study was assessed qualitatively rather than through standardized quantitative measurements. Therefore, correlation analysis between graft thickness and clinical outcomes was not performed to avoid potential bias. In addition, no clear deterioration in short-term clinical outcomes was observed despite these imaging findings. Taken together, graft thinning observed in this study may reflect early biological remodeling or partial structural compromise, and its clinical significance remains uncertain. Further studies with quantitative imaging protocols and longer follow-up are required to determine whether graft thinning is predictive of long-term failure or functional decline.

The use of the PLT as an autograft represents a relatively recent option for SCR. Several studies have demonstrated its utility as an adjunct to lower trapezius transfer in the treatment of IRCTs (Bozoğlan et al., 2023; Sundararajan et al., 2025; Elhalawany et al., 2025; Rajagopalan et al., 2025; Kuberakani et al., 2025). Regarding the application of PLT as an SCR graft, Li et al. proposed a technique involving the creation of a bone tunnel at the glenoid, which may increase surgical complexity (Li et al., 2023). Subsequent cadaveric biomechanical studies and *in vivo* rabbit models validated this approach (Wang et al., 2025a; Peng et al., 2025; Wang et al., 2025b). The results indicated favorable outcomes in preventing superior migration of the humeral head, reducing subacromial contact pressure, and promoting tendon-to-bone healing. Their clinical follow-up study further reported satisfactory functional outcomes, with 94.4% of patients showing good healing at 1 year postoperatively (Wang et al., 2025c). In another study, PLT was employed to augment LHBT transposition. The combined use of PLT and LHBT transposition was associated with lower retear rates and better abduction function compared with LHBT transposition alone (Zhou et al., 2025). Our strategy has its advantages and disadvantages: First, the length of the PLT implant is adjustable based on the requirement during the operation, eliminating the need for intra-articular measurement. Second, the PLT is relatively easy to harvest and less traumatic, requiring merely a small incision. Third, the procedure does not involve the application of new instruments, making it relatively easy to replicate. However, the surgery duration is still long. The management of sutures and anchors is also time-consuming and complex.

This study has the following limitations. First, this study is absent of cyclic loading, fatigue testing, and other advanced mechanical analyses. Future studies incorporating dynamic and failure testing will be necessary to further evaluate the durability and mechanical behavior of this technique. Second, removing only the supraspinatus tendon does not fully replicate the complexity of posterosuperior massive tears involving both the supraspinatus and infraspinatus. This simplified model was selected to ensure reproducibility and consistency across specimens and to isolate the effect of superior capsule reconstruction on superior stability. However, this may limit direct clinical translation. Third, this study did not include a control group using alternative treatment strategies, such as fascia lata autografts, dermal allografts, or reverse shoulder arthroplasty. Therefore, no conclusions can be drawn regarding the superiority or equivalence of the proposed technique compared with existing surgical options. The present study should be interpreted as a feasibility and preliminary effectiveness investigation rather than a comparative study. Forth, although this study included only 9 patients, the repeated-measures design and the large observed improvement in ASES score supported adequate power for the primary endpoint; however, the study may have been underpowered for smaller changes in secondary outcomes.

## 5 Conclusion

SCR with AT transplantation may provide a viable surgical option for the management of IRCTs by improving superior stability of the glenohumeral joint under experimental conditions. Preliminary clinical findings suggest potential functional improvement; however, due to the limited sample size, absence of a control group, and relatively short follow-up, the results should be interpreted with caution. Further well-designed comparative studies are needed to validate its clinical efficacy and determine its role among existing treatment strategies.

## Data Availability

The raw data supporting the conclusions of this article will be made available by the authors, without undue reservation.
